# Exposure to Particulate Matter (PM) During Pregnancy and Non-Hodgkin Lymphomas in Children

**DOI:** 10.3390/cancers18172746

**Published:** 2026-08-24

**Authors:** Gema Monteagudo, Lidia Pérez Ormita, Mònica Guxens, Adela Cañete, Elena Pardo Romaguera, Diana Gómez-Barroso, María Alonso-Colón, Beatriz Núñez-Corcuera, Juan Antonio Ortega García, Rebeca Ramis

**Affiliations:** 1 UNED-ENS Mixed Research Institute (IMIENS), 28029 Madrid, Spain; gmonteagu10@alumno.uned.es (G.M.);; 2 Rey Juan Carlos University Hospital, 28933 Móstoles, Spain; 3 IS-Global, 08003 Barcelona, Spain; 4 ICREA, 08003 Barcelona, Spain; 5 Departament MELIS-UPF, Universitat Pompeu Fabra, 08005 Barcelona, Spain; 6 Centre for Biomedical Research in Epidemiology & Public Health (CIBER Epidemiología y Salud Pública-CIBERESP), 28029 Madrid, Spain; 7 Department of Child and Adolescent Psychiatry/Psychology, Erasmus MC, University Medical Centre, P.O. Box 2060, 3000 CD Rotterdam, The Netherlands; 8 Spanish Registry of Childhood Tumours (RETI-SEHOP), University of Valencia, 46010 Valencia, Spain; adela.canete@uv.es (A.C.); elena.pardo@uv.es (E.P.R.); 9 National Center for Epidemiology, Instituto de Salud Carlos III (Carlos III Institute of Health), 28029 Madrid, Spain; 10 National Center for Environmental Health, Instituto de Salud Carlos III (Carlos III Institute of Health), 28220 Madrid, Spain; b.nunez@isciii.es; 11 Pediatric Environmental Health Speciality Unit (Pehsu), Hospital University Virgen Arrixaca, 30120 Murcia, Spain; 12 IMIB-Pascual Parrilla, 30120 Murcia, Spain; 13 Pediatrics Area, Department of Surgery, Pediatrics, Obstetrics and Gynecology, Faculty of Medicine, Health Sciences Campus, University of Murcia, Avda. Buenavista, 32, El Palmar, 30120 Murcia, Spain

**Keywords:** PM_2.5_, PM_10_, environmental exposure, childhood cancer, non-Hodgkin lymphoma, incidence, epidemiology

## Abstract

**Background/Objectives**: Lymphomas are the third most common pediatric malignancy, with non-Hodgkin lymphoma (NHL) comprising around 7% of childhood cancers. Environmental exposures have been linked to its etiology. Particulate matter (PM) prenatal exposure is increasingly scrutinized for potential effects on early-life carcinogenesis. We investigated whether exposure during pregnancy to PM_2.5_ and PM_10_ was associated with childhood NHL incidence. **Methods**: A population-based case–control study was conducted in Spain (2009–2016), including 3,682,038 children < 15 years. Incident NHL cases (n = 289) were identified through the Spanish Childhood Tumor Registry. Prenatal PM_2.5_ and PM_10_ exposures were estimated using a random forest model. Logistic regression estimated odds ratios (ORs) and 95% confidence intervals (CIs). Exposures were modeled continuously and categorically by tertiles (low, medium, or high exposure) to assess nonlinearity; trimester-specific analyses were also explored. **Results**: In the analysis with the continuous exposure, higher prenatal PM_2.5_ was associated with increased odds of NHL, and PM_10_ showed smaller associations. With categorical variables, medium and high-level exposure to PM_2.5_ showed an association with higher NHL incidence, especially in the third trimester. High-level exposure to PM_10_ during the first and second trimesters was linked to a higher NHL incidence in children under five years. **Conclusions**: Maternal PM_2.5_ and PM_10_ exposure during pregnancy could be associated with childhood NHL incidence, with stronger associations among children diagnosed before age 5. These findings warrant replication and mechanistic investigation.

## Simple Summary

Lymphomas are the third most frequent tumors in children under 15 years old, with non-Hodgkin lymphoma (NHL) comprising around 7% of childhood cancers. Lymphomas have few known risk factors, such as infectious agents, immune disorders, and environmental exposure to pollutant agents. This paper aims to better understand childhood lymphomas’ etiopathogenesis by analyzing the effect of prenatal particulate matter exposure on the incidence of NHL in children. We conducted a population-based case-control study with data from Spain from 2009 to 2016. We estimated the concentrations of PM_10_ and PM_2.5_ for the entire pregnancy by a spatiotemporal random-forest model. Logistic regressions were modeled, and odds ratios (ORs) and 95% confidence intervals (CIs) calculated. Our findings suggest a potential association between exposure to PM_2.5_ during pregnancy and the risk of developing NHL in children.

## 1. Introduction

In 2023, the number of estimated deaths among children 0–14 years due to cancer worldwide was 107,200, as it was the third main cause, accounting for 65,000 deaths among children 5–14 years [1,2]. Same-year estimates for Europe reach 3280 deaths related to cancer, meaning that cancer was the main cause of death among children 5–14 years and the sixth cause for children under five years [1,2]. In Spain, the number of cancer-related deaths in children 0–14 years was 172 [1,2]. The same year, European estimates for Spain reached 993 new cases of cancer for children aged 0–14 years [3]. The top three types of cancer were leukemia, central nervous system (CNS), and lymphomas, and they together account for two out of three cancer diagnoses in children [3,4]. Together, non-Hodgkin lymphoma and Burkitt lymphoma represented 57.1% of lymphomas registered in children in Spain from 2010 to 2023 [3].

The International Consensus Classification (ICC) organizes lymphoid neoplasms according to cell immunophenotype (B or T cell lymphomas) and to maturation stage (precursor or mature cell lymphomas) [5]. NHL refers to lymphoid malignancies derived from B or T cell progenitors or mature cells and usually shows an aggressive clinical behavior in children [6]. NHL incidence rates on the European continent range from 6.4 per million in Ukraine to 12.2 per million in Spain [7]. In recent years, a rise in NHL incidence rates has been described and connected to environmental factors [8].

In big cities and near industrial areas, ambient air contains a wide variety of known carcinogens such as polycyclic aromatic hydrocarbons, dioxins, benzene, and particles. Particulate matter (PM) includes small particles of sulfate, nitrates, ammonia, black carbon, organic compounds, and mineral dust, among others, and most of them are products of traffic emissions that are suspended in the air [9,10]. PM can be classified by diameter in PM_2.5_ (particles with a diameter of 2.5 micrometers or less) and PM_10_ (10 micrometers or less) [9]. The pollutant responsible for most air pollution-related deaths is PM, which has been classified as a Group 1 carcinogen by the International Agency for Research on Cancer (IARC) [6,7]. Long-term exposure to PM_2.5_ has been associated with childhood cancer in previous studies [10]. PM_2.5_ has been specifically linked to lung, breast, liver, esophageal cancer, or leukemia [11,12]. Some studies have found that residing close to industrial areas could increase the risk of cancer, specifically NHL [13,14,15]. A Danish study found an association between PM_2.5_ exposure in childhood and NHL development, particularly related to its black carbon component [16].

Environmental pollutants can interact with other factors such as genetic predisposition, immune diseases, or infectious agents in childhood cancer development, including in utero exposure [17]. This is possible because, in the case of PM, it can cross the placental barrier and reach fetal blood [13]. PM_2.5_ has shown potential to damage placenta structure via mitochondrial dysfunction and systemic inflammation due to reactive oxygen species (ROS) [18,19].

Few studies have specifically examined the relationship between air pollution and childhood NHL, and the available evidence remains limited and heterogeneous. Prenatal exposure may be particularly relevant, as particulate matter can cross the placental barrier and may affect fetal development through oxidative stress, inflammation, and placental dysfunction. In this study, we evaluated whether prenatal exposure to PM_2.5_ and PM_10_ was associated with childhood NHL in Spain.

## 2. Materials and Methods

### 2.1. Study Design

We conducted a population-based epidemiological study in Spain including 3,682,038 children born in the period 2004–2016.

Cases were children under 15 years old with a diagnosis of NHL from 14 autonomous communities before 2022. These registries were retrieved from the Spanish Registry of Childhood Tumor (RETI-SEHOP), a collaborative project between the Spanish Society of Pediatric Hematology and Oncology and Valencia University, which collects data from hospitals [20]. The RETI-SEHOP database encodes incidence cancer cases using the International Classification of Childhood Cancer (ICCC-3.1) [21]. For this study, we selected cases of NHL corresponding to ICCC-3 codes IIb (non-Hodgkin lymphomas, except Burkitt lymphoma), IIc (Burkitt lymphoma), and IId (miscellaneous lymphoreticular neoplasms). We gathered a total of 289 diagnosed children born between 2004 and 2016 that developed cancer up to 2021. This meant that those children born at the beginning of the study, in the years 2004–2007, had enough study time to turn 15 years old, while those born later did not. Incident NHL cases diagnosed later than 2021 were not included, and, therefore, they did not account for our analysis because their data were not available in the cancer registry when this study was conducted.

The control population involved newborns in the studied regions and period with available residential data, and data was supplied by the Spanish Official Birth Registry from the National Statistics Institute (INE) [22]. In total, 3,681,749 children were included as controls.

The study regions included the following Autonomous Communities: Andalusia, Asturias, Aragon, Cantabria, Castilla-La Mancha, Castilla y León, Catalonia, the Valencian Community, Extremadura, Galicia, La Rioja, Madrid, Murcia, Navarre, the Basque Country, and the Balearic Islands. Though the Canary Islands, Ceuta, and Melilla were not included in the study because exposure measures were not available for these regions.

### 2.2. Air Pollution Concentration Measures

To estimate prenatal exposure to PM_2.5_ and PM_10_, we used the geographical coordinates of the mother’s residence during pregnancy facilitated by RETI-SEHOP and INE, and we assumed they did not change residency during pregnancy. PM_2.5_ and PM_10_ estimated concentrations by geographical areas were calculated with spatiotemporal land use random forest models. These models link ground-level air pollution and satellite-based measures of aerosol optical depth, land use, meteorological conditions, and traffic variables to estimate PM over 1 km × 1 km grid cells and were developed by Stafoggia to estimate daily PM_10_ and PM_2.5_ concentrations in Italy [23]. We applied this methodology in a previous study on PM exposure during pregnancy and birth outcomes, where more information on the specific model was presented [24]. For the present study, we used the same estimates corresponding to PM_10_ and PM_2.5_ daily concentrations for the entire pregnancy period at the maternal address at the time of birth. Then, we computed the average exposure for the total pregnancy and by trimester. PM_10_ estimations were made with data for 2004–2016, and PM_2.5_ estimations were made with 2009–2016 data, as the corresponding air quality monitoring networks were set up in 2004 and 2009, respectively. Because of these two periods studied, we would refer to two different exposed populations: one for PM_10_, born in the period 2004–2016, and a second one for PM_2.5_, born in the period 2009–2016.

### 2.3. Potential Confounding Variables

We included resource deprivation at the census track level and population size of the municipality as confounding variables. Deprivation was measured with an index that ranges from lower (−2.58) to higher (4.88) deprivation regarding six indicators: manual and temporary workers, unemployment, insufficient education overall, in young people between 16 and 29 years old, and no internet access [25]. The population size of the municipality of the mother’s residence was categorized as follows: equal to or less than 10,000 inhabitants, 10,001–20,000 inhabitants, 20,001–50,000 inhabitants, 50,001–100,000 inhabitants, more than 100,000 inhabitants, and towns that are the capital city of a province.

### 2.4. Statistical Analysis

For the analysis, exposure to PM_2.5_ and PM_10_ were considered both as a continuous variable (assuming linear effects) and as a categorical variable with three levels: low (reference category), medium, and high levels of exposure to PM. For each studied population and exposure window (population exposed to PM_2.5_ and population exposed to PM_10_), we defined the categories as the tertiles (0–0.33, 0.33–0.66, 0.66–1) of the distribution of the exposure variable. Estimated exposure information was measured in μg/m^3^ units.

We conducted analyses for the entire pregnancy period, specific trimesters, age, the entire study population (cases < 15 years old), and the group of children under five years. To assess the association between PM exposure during pregnancy and NHL, we used logistic regressions and calculated odds ratios (ORs) and 95% confidence intervals (CIs). To evaluate the non-linear relationship between PM levels and NHL incidence, we created generalized additive models (GAM) graphics. A *p*-value < 0.05 was set as statistically significant. Analyses were made using R software, Version 4.4.1.

Models were adjusted by sex, year of birth, autonomous community, deprivation level, and size of municipality.

## 3. Results

### 3.1. Descriptive Analysis

The descriptive analysis shows the distribution of the studied population by sex, mean age at diagnosis, deprivation index, and PM exposure and is presented in Table 1. As we had two different periods of exposure data for PM_10_ and PM_2.5_, we included two descriptive sections in the table. For the population exposed to PM_10_, the control group comprised the source population excluding cases; the characteristics of the total population and the controls were nearly identical. The cohort included 48% of females and 52% of males; its mean deprivation index was −0.29, and its PM_10_ total pregnancy mean was 25.13 μg/m^3^. For both sets of cases, those under 15 and under 5 years old showed different sex ratios, with 31% female and 69% male. The mean age at diagnosis was 6.06 years for the under-15 group and 2.74 for the under-5. Regarding the deprivation index, cases showed more deprivation; PM_10_ exposure cases under 15 showed higher exposure, but cases under 5 showed less exposure than the controls.

The population exposed to PM_2.5_ is smaller than PM_10_ because of the shorter studied period. Descriptive results for the sex ratio are basically the same. Cases were younger on average than those in the exposed to PM_10_ population. The deprivation index showed a slightly higher mean, with cases showing more deprivation than controls. And regarding PM_2.5_, the mean for cases was higher than for controls.

### 3.2. Logistic Regression

Table 2 shows the results from logistic regressions performed for children under 15 years and under 5 years. In models with a continuous exposure variable, PM_2.5_ prenatal exposure during the entire pregnancy period was associated with an 8% increase in odds of NHL (OR = 1.08; 95% CI [0.98–1.19]) for children under 15 years and 14% for the group under 5 years (OR = 1.14: 95% CI [1.00–1.21]). PM_10_ also showed an association with OR = 1.04 (95% CI [0.99–1.08]) for children under 15 years, and OR = 1.03 (95% CI [1.00–1.04]) for children under 5 years. In both cases, only the association for children under 5 years was statistically significant.

Regarding PM levels as a categorical variable during the pregnancy period, results for PM_2.5_ showed 71% higher odds of NHL for the medium exposure level (OR = 1.71; 95% CI [1.06–2.05]) and 2.5 times the odds for the high exposure level (OR = 2.53; 95% CI [1.43–4.50]) compared to the low exposure level for children under 15 years. For children under 5 years, the association found for the medium exposure level was a 95% increase in odds (OR = 1.95; 95% CI [1.00–2.75]) and almost four times for the highest exposure level (OR = 3.99; 95% CI [1.82–8.73]) compared to the low exposure level. Exposure to PM_10_ during the pregnancy period was associated with higher NHL odds only for the high exposure levels with a 59% increase (OR = 1.59; 95% CI [1.07–2.01]) in children under 5 years.

For PM_2.5_, when these analyses were conducted separately by trimester of pregnancy, no association was found for the continuous variable, but for the high exposure level compared to the low exposure level for the third trimester in both children under 15, the data showed a 72% increase in odds (OR = 1.72; 95% CI [1.04–2.71]), and for those under 5 years, the increase was 2.5 times (OR = 2.55; 95% CI [1.29–4.50]). For PM_10_ exposure in children under 15 years, it showed an association with NHL in the first and second trimesters of pregnancy for high exposure levels compared to low exposure level with 74% increased odds for the first trimester (OR = 1.74; 95% CI [1.14–2.66]) and 59% increased odds for the second trimester (OR = 1.59; 95% CI [1.03–2.45]). And for children under 5 years, ORs were more than twice the odds (OR = 2.17; 95% CI [1.07–4.41]) for the second trimester.

Results from trimester-specific models, continuous and categorical, showed, in general, lower ORs in the third trimester. However, for PM_2.5_, the ORs for the third trimester were the highest, and their estimations were statistically significant.

Finally, Table 3 shows the results from logistic regressions performed for the subtypes corresponding to ICCC-3 codes 22 (Non-Hodgkin lymphomas, except Burkitt lymphoma) and 23 (Burkitt lymphoma) for children under 15 years and for exposure during the entire pregnancy. For both subgroups, the estimated ORs suggested higher incidence but with lower values than those in Table 2, where all NHLs were together. ORs for code 22 were higher than ORs for Burkitt lymphoma.

### 3.3. Non-Linear Analysis

**Figure 1:**
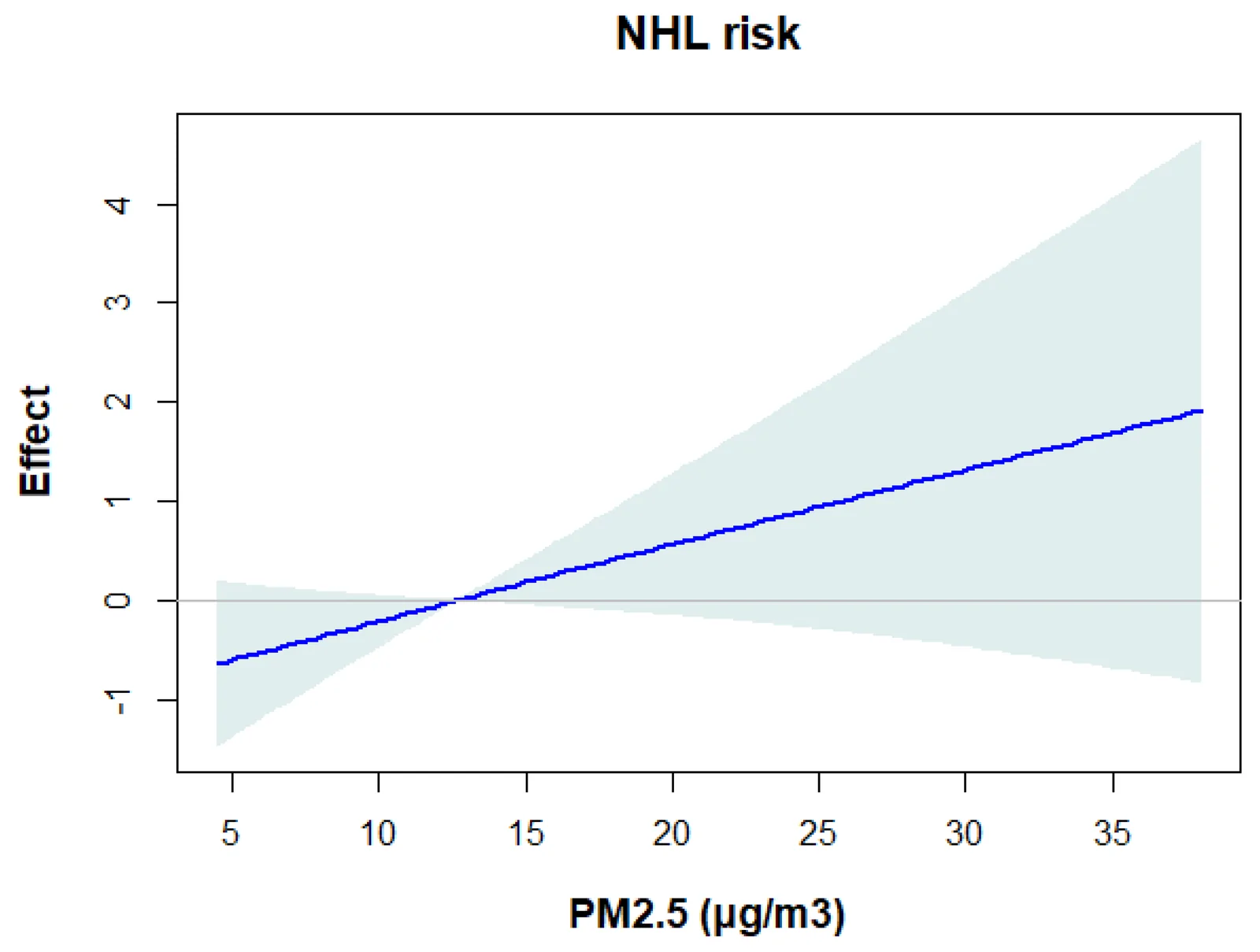
Relationship between PM_2.5_ exposure for the entire pregnancy in μg/m^3^ and NHL risk. The solid line represents the estimated effect of PM_2.5_ exposure on NHL risk. The shaded area represents the 95% CI.

**Figure 2:**
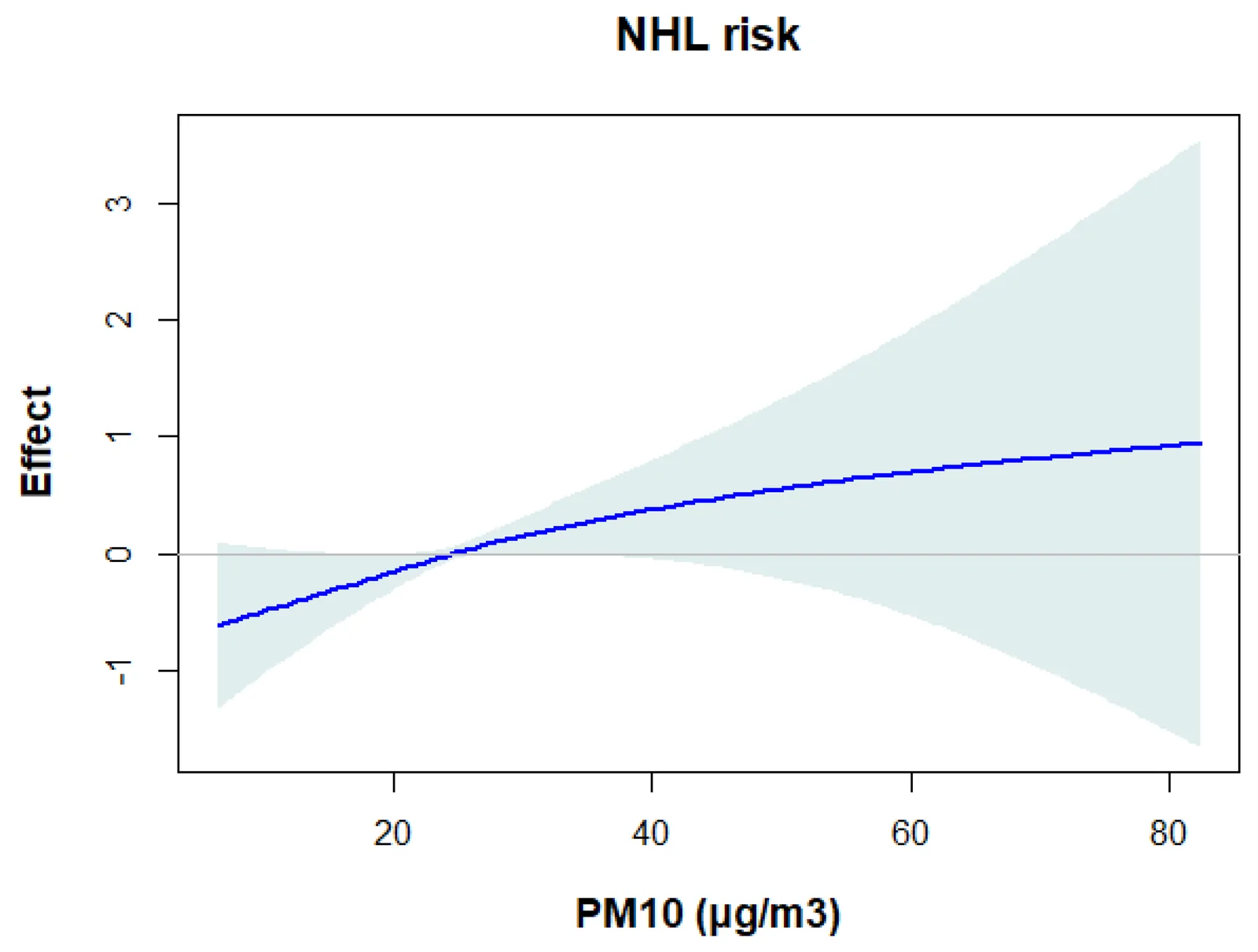
Relationship between PM_10_ exposure for the entire pregnancy in μg/m^3^ and NHL risk. The solid line represents the estimated effect of PM_10_ exposure on NHL risk. The shaded area represents the 95% CI.

Figure 1 and Figure 2 show GAM graphics evaluating the potential non-linear relationship between each type of PM exposure for the entire pregnancy period and NHL incidence, where the *y*-axis represents the proportional effect in estimated NHL risk with a change in PM exposure relative to its mean value. The graphic for PM_2.5_ exposure showed almost a linear relationship with NHL risk, indicating a possible dose-response relationship when PM_2.5_ rises above 13 μg/m^3^.

As for PM_10_ exposure, the GAM graphic shows a non-linear trend whereby the risk appears to increase from around 25 μg/m^3^ up to approximately 50 μg/m^3^ and then the slope seems to flatten.

## 4. Discussion

In this study, we investigated the potential association between exposure to PMs during pregnancy and NHL incidence in children. Our results support the hypothesis of increasing incidence in those exposed to higher levels of PMs, both PM_2.5_ and PM_10_. Findings from continuous, categorical, and non-linear analysis showed an increased incidence that was stronger among pregnant women exposed to the highest levels. We observed a higher incidence of NHL, particularly in children under 5 years old who were exposed to high levels of PM_2.5_ for the entire pregnancy period. Regarding the exposure window by gestational trimester, findings were unclear; conversely, for the non-linear analysis, the results suggested a potential dose-response relationship. Analysis of the lymphoma subtype did show a lower incidence of Burkitt than NHL (code IIb in ICCC-3.1).

In our study, most of the NHL cases were more likely to be male for both types of PM exposure, as in various studies [26,27,28]. Average PM exposure during pregnancy was higher in the cases compared to the controls, but without statistical significance, other studies did not show differences in PM exposure levels [26,29]. In the study by Hvidtfeldt et al., cases resided in neighborhoods with a low proportion of manual workers, and in our study, cases seemed to occur in areas with higher scores of deprivation index, which reflects a greater proportion of manual workers [27].

The findings in our study are consistent with previous investigations like the Williams et al. study, carried out in children under 16 in Texas, where they found cancer cases lived close to areas with higher PM_2.5_ levels, and PM_2.5_ was statistically associated with a 24% excess risk for NHL [26]. This study evaluated exposure during the pre-conception period, in utero, and postnatal period, unlike ours, where only pregnancy period exposure was assessed because we did not have postnatal information available due to the lack of residential history for cases and controls.

The study by Hvidtfeldt et al. analyzed different sub-components of PM_2.5_ and their association with NHL in children under 20 years old in Denmark. They found an association between postnatal PM_2.5_ exposure and NHL incidence; in addition, they suggested that this relationship may be due to the black carbon sub-component, a hypothesis also formulated in a previous study carried out in Denmark by Taj. et al. in adult participants [16,28].

The study by Lavigne et al. about maternal exposure to air pollutants and early childhood cancers in Canada showed an association for PM_2.5_ exposure during the first trimester and an increased risk of astrocytoma in childhood, but not with other cancers [28].

Other studies showed different results compared to ours, for example, Clark et al. The study analyzed prenatal exposure to PM_2.5_ and its relationship with childhood cancer in Minnesota, and they found no association between PM_2.5_ exposure and the risk for all cancers considered (including NHL), but individual analysis showed that PM_2.5_ exposure during the first trimester of pregnancy was associated with increased odds of Burkitt Lymphoma [29].

Another study analyzed the association between air pollutant exposure during pregnancy, including PM_10_, and the risk of childhood cancer in Israel, carried out by Farhi et al. They found no significant association between the exposure and overall childhood cancer, but, as they argue in their discussion, they had few cases (453 cancer cases) compared to the entire sample size (216,730 children) and only analyzed exposure for the overall pregnancy period and the first trimester [30].

The heterogeneity of the results from different studies can be explained by different methodologies used in the exposure estimation, the statistical analysis, the number of pollutants studied each time, or the adjustment for specific covariates, among others. Some studies, like the present paper, focused on NHL [27]. Others studied various types of cancer, depending on their objectives and data availability [16,29,30]. Moreover, like our study, some investigations studied only one air pollutant at a time, with PM_2.5_ as the main object of study [16,29]. In contrast, some studies tried to find associations for various contaminants [28,30].

Concerning dose-response analysis, few studies assessed the dose–response relationship between air pollutants and childhood cancer, but they did not evaluate PM and were focused on leukemia in most cases [31,32]. A study conducted in Canada assessed ultrafine particles (UFPs) and their association with childhood cancer incidence (including NHL) and found a linear relationship between UFP exposure during the first trimester and childhood cancers, like our finding of an almost linear relationship between PM_2.5_ and NHL risk [33]. Although they did not evaluate PM 2.5 exposure individually, they found a weak correlation for UFPs and PM_2.5_ [33].

Major differences among studies are the covariates or confounding factors accounted for in their analysis. A limitation of our study is that we did not have individual data on parents’ medical records, occupational or environmental exposures, or lifestyle factors; therefore, we only addressed sex, year of birth, deprivation index, size of municipality, and autonomous community as covariates to adjust our models. Also, we lacked information about other sociodemographic aspects as we had no other data available. Some studies included socio-economic status (SES) in their analysis [16,26,28]. For example, the study from Williams et al. included data about maternal race and ethnicity [25], or the study by Hvidtfeldt et al. had information about birth weight, parental education, or maternal exposure to tobacco smoke [16].

In reference to the exposure estimation methodology, only one study carried out in Israel by Farhi et al. used exposure data based on measurements from air-quality monitoring stations [31], while the rest of the studies estimated exposure to air pollutants by using different modeling methods, as done in the present paper [16,26,28,29].

Another limitation of our study is the different follow-up period for each participant, since the final year for the diagnosis was 2021. As we mentioned in the Section 2, children born at the beginning of the study, years 2004–2007, had enough follow-up time to turn 15 years old, while those born later did not have enough follow-up time. This issue may have led us to underestimate NHL incidence in our sample, as the median age at diagnosis is about 10 years old and cases diagnosed after 2021 were not collected [6]. A related issue is that we assessed pregnancy exposure only, not including postnatal exposure to PM. With postnatal exposure data available, we could have found a higher OR for NHL incidence, not only in the group of children under 5 years old but also in children under 15 years old, as Williams et al. did in Texas [26].

A shared limitation of previous studies and ours is the fact that we assumed that mothers did not change their residence during the pregnancy period, and, in cases where this was not true, it could have led to PM exposure misclassification [16,26,28,30]. A review of the medical literature assessed exposure misclassification in studies about environmental exposure and found that 9–32% of mothers moved during pregnancy, especially in the second trimester, also considering that moves during early pregnancy are more likely to influence exposure estimates. However, the median distance moved was less than 10 km, as most mothers stayed in the same area and in the same country; therefore, most moves did not greatly change exposure estimates [34]. Also, residual bias due to unequal follow-up across birth cohorts cannot be excluded.

The quality of our data is a strong point, as we used data collected by nationally validated data sources such as INE for the Birth Registry or RETI-SEHOP for all NHL cases diagnosed in children in Spain over 40 years [20]. The fact that our study covered practically the entire Spanish territory except for the Canary Islands, Ceuta, and Melilla helped us to approximate the real situation regarding NHL incidence and exposure to PM_10_ and PM_2.5_ in Spain. Another strength of this study is the availability of individual PM exposure data calculated by validated methods such as random forest models.

Finally, we should mention that the maximum levels of PMs are legally established by the corresponding authorities. The levels of PM_2.5_ and PM_10_ estimated in this study did not exceed the current legal health limits set for annual exposure for Spain [35]. However, the new EU Directive 2024/2881 of the European Parliament is more restricted and establishes lower limits to be reached by January 2030; some of our higher PM estimates would exceed these new limits [36]. In contrast, the World Health Organization (WHO) air quality guidelines [37] set even lower limits that are closer to the non-effect values identified in this study.

## 5. Conclusions

The results of this study suggest a potential association between prenatal exposure to PM_2.5_ and PM_10_ and an increased risk of NHL in children. The findings also point to a potential dose–response relationship, in which higher exposure levels are linked to greater incidence, although no specific critical exposure window was identified. In this context, an elevated incidence of childhood NHL was observed when PM_2.5_ exposure exceeded 13 μg/m^3^ and PM_10_ exposure exceeded 30 μg/m^3^. These results suggest that current World Health Organization (WHO) air quality guidelines may warrant reconsideration. From a public health perspective, efforts to reduce air pollution could contribute to lowering the incidence of NHL in children.

Only a few studies focus on PM and its relationship with NHL in children with heterogeneity. Therefore, more studies should be conducted to better understand the PM role in NHL development in children, warranting replication and mechanistic investigation.

## Figures and Tables

**Table 1:** Descriptive values of the sample, including percentages with frequencies of individuals’ sex ratio and mean with its standard deviation (SD) for the remaining characteristics.

Population Exposed to PM_10_, Birth Period 2004–2016				
Characteristics	Total	Control	NHL < 15 years old	NHL < 5 years old
Total	3,682,038	3,681,749	289	101
Female	1,781,636 (48%)	1,781,547 (48%)	89 (31%)	32 (32%)
Male	1,900,402 (52%)	1,900,202 (52%)	200 (69%)	69 (68%)
Mean age at diagnosis in years (SD)	-	-	6.06 (3.5)	2.47 (1.40)
Deprivation (SD) ^1^	−0.29 (0.94)	−0.29 (0.94)	−0.40 (0.93)	−0.40 (0.96)
PM_10_ total pregnancy mean (SD) ^1^	25.13 (6.63)	25.13 (6.63)	26.60 (6.57)	24.59 (6.51)
**Population Exposed to PM_2.5_, Birth Period 2009–2016**				
Characteristics	Total	Control	NHL < 15 years old	NHL < 5 years old
Total	1,994,837	1,994,704	133	71
Female	966,422 (48%)	966,381 (48%)	41 (31%)	22 (31%)
Male	1,028,415 (52%)	1,028,323 (52%)	92 (69%)	49 (69%)
Mean age at diagnosis in years (SD)	-	-	4.53 (2.53)	2.42 (1.47)
Deprivation (SD) ^1^	−0.23 (0.98)	−0.23 (0.98)	−0.37 (0.99)	−0.36 (0.95)
PM_2.5_ total pregnancy mean (SD) ^1^	9.30 (2.18)	9.30 (2.18)	9.88 (2.84)	9.54 (2.29)

^1^ Non-significant differences in the *t*-test.

**Table 2:** Logistic regression results. Levels of PM for the categories in μg/m^3^. ORs, their respective 95% CIs, and *p*-values.

Under 15 Years Old Population								
	Total Pregnancy				First Trimester			
PM_2.5_	Levels	OR	95% CI	*p*-Value	Levels	OR	95% CI	*p*-Value
**Continuous**		1.08	(0.98, 1.19)	0.11		1.04	(0.96, 1.12)	0.36
**Low (ref)**	<11.43	1			<11.39	1		
**Medium**	11.43–13.36	1.71	(1.06, 2.75)	0.03	11.39–13.61	1.32	(0.85, 2.07)	0.22
**High**	>13.36	2.53	(1.43, 4.50)	0.01	>13.61	1.33	(0.78, 2.26)	0.29
**PM_10_**	**Levels**	**OR**	**95% CI**	***p*** **-value**	**Levels**	**OR**	**95% CI**	***p*** **-value**
**Continuous**		1.03	(1.00, 1.06)	0.06		1.02	(0.99, 1.04)	0.15
**Low (ref)**	<21.40	1			<21.41	1		
**Medium**	21.40–27.57	1.16	(0.81, 1.65)	0.42	21.41–27.90	1.23	(0.87, 1.73)	0.25
**High**	>27.57	1.71	(1.07, 2.71)	0.02	>27.90	1.74	(1.14, 2.66)	0.01
	**Second trimester**				**Third trimester**			
**PM_2.5_**	**Levels**	**OR**	**95% CI**	***p*** **-value**	**Levels**	**OR**	**95% CI**	***p*** **-value**
**Continuous**		1.07	(0.98, 1.16)	0.12		1.06	(0.98, 1.14)	0.13
**Low (ref)**	<11.30	1			<11.22	1		
**Medium**	11.30–13.40	1.37	(0.88, 2.15)	0.17	11.22–13.31	1.19	(0.75, 1.89)	0.46
**High**	>13.40	1.47	(0.86, 2.50)	0.16	>13.31	1.72	(1.04, 2.56)	0.04
**PM_10_**	**Levels**	**OR**	**95% CI**	***p*** **-value**	**Levels**	**OR**	**95% CI**	***p*** **-value**
**Continuous**		1.03	(1.00, 1.05)	0.04		1.02	(0.99, 1.04)	0.19
**Low (ref)**	<21.25	1			<21.07	1		
**Medium**	21.25–27.53	1.27	(0.88, 1.74)	0.23	21.07–27.18	1.19	(0.88, 1.61)	0.25
**High**	>27.53	1.59	(1.03, 2.45)	0.04	>27.18	1.28	(0.89, 1.85)	0.19
**Under 5 years old population**								
	**Total pregnancy**				**First trimester**			
**PM_2.5_**	**Levels**	**OR**	**95% CI**	***p*** **-value**	**Levels**	**OR**	**95% CI**	***p*** **-value**
**Continuous**		1.14	(1.00, 1.29)	0.04		1.08	(0.97, 1.19)	0.16
**Low (ref)**	<11.97	1			<11.38	1		
**Medium**	11.97–12.29	1.95	(1.00, 3.80)	0.05	11.38–13.61	1.73	(0.93, 3.21)	0.08
**High**	>12.29	3.99	(1.82, 8.73)	0.01	>13.61	1.88	(0.90, 3.91)	0.09
**PM_10_**	**Levels**	**OR**	**95% CI**	***p*** **-value**	**Levels**	**OR**	**95% CI**	***p*** **-value**
**Continuous**		1.04	(0.99, 1.09)	0.1		1.03	(0.99, 1.07)	0.15
**Low (ref)**	<21.40	1			<21.41	1		
**Medium**	21.40–27.57	1.24	(0.66, 1.97)	0.64	21.41–27.90	1.57	(0.83, 2.65)	0.09
**High**	>27.27	1.87	(0.86, 4.05)	0.11	>13.61	1.83	(0.99, 3.78)	0.1
	**Second trimester**				**Third trimester**			
**PM_2.5_**	**Levels**	**OR**	**95% CI**	***p*** **-value**	**Levels**	**OR**	**95% CI**	***p*** **-value**
**Continuous**		1.11	(1.00, 1.23)	0.05		1.09	(0.98, 1.20)	0.1
**Low (ref)**	<11.30	1			<11.22	1		
**Medium**	11.30–13.40	1.38	(0.74, 2.56)	0.31	11.22–13.31	1.23	(0.64, 2.38)	0.53
**High**	>13.40	1.75	(0.85, 3.60)	0.13	>13.31	2.55	(1.29, 5.06)	0.01
**PM_10_**	**Levels**	**OR**	**95% CI**	***p*** **-value**	**Levels**	**OR**	**95% CI**	***p*** **-value**
**Continuous**		1.05	(1.01, 1.09)	0.02		1.02	(0.97, 1.05)	0.68
**Low (ref)**	<21.25	1			<21.07	1		
**Medium**	21.25–27.53	1.12	(0.65, 1.93)	0.69	21.07–27.18	0.98	(0.59, 1.63)	0.94
**High**	>13.40	2.17	(1.07, 4.41)	0.03	>13.31	1.56	(0.81, 2.98)	0.18

Statistical significance was defined as *p*-value < 0.05.

**Table 3:** Logistic regression results for lymphoma subtypes 22 and 23. Levels of PM for the categories in μg/m^3^. ORs, their respective 95% Cis, and *p*-values.

<15 Years		Non-Hodgkin Lymphomas, Except Burkitt Lymphoma, ICCC-3 Code = 22				Burkitt Lymphoma, ICCC-3 Code = 23			
		55 Cases Exposed to PM_2.5_ 135 Cases Exposed to PM_10_				57 Cases Exposed to PM_2.5_ 130 Cases Exposed to PM_10_			
**PM_2.5_**		**Levels**	**OR**	**95% CI**	**P1**	**Levels**	**OR**	**95% CI**	**P1**
	**Continuous**		1.08	(0.94, 1.25)	0.28		1	(0.86, 1.17)	0.97
	**Low (ref)**	<11.41	1			<11.41	1		
	**Medium**	11.41–13.31	1.11	(0.52, 2.36)	0.8	11.41–13.32	1.1	(0.54, 2.65)	0.8
	**High**	>13.31	2.07	(0.88, 4.89)	0.1	>13.32	2.09	(0.88, 4.94)	0.09
**PM_10_**		**Levels**	**OR**	**95% CI**	**P1**	**Levels**	**OR**	**95% CI**	**P1**
	**Continuous**		1.04	(1.00, 1.08)	0.05		1	(0.97, 1.06)	0.49
	**Low (ref)**	<21.39	1			<21.39	1		
	**Medium**	21.39–27.57	1.16	(0.67, 2.01)	0.59	21.39–27.57	1.05	(0.62, 1.78)	0.87
	**High**	>27.57	2.24	(1.13, 4.43)	0.02	>27.57	1.35	(0.68, 2.69)	0.39

## Data Availability

Restrictions apply to the availability of these data. Data were obtained from the Spanish National Statistics Institute (INE) www.ine.es (accessed on 31 March 2025).

## Abbreviations

The following abbreviations are used in this manuscript:

CNS	Central nervous system
NHL	Non-Hodgkin lymphoma
ICC	International Consensus Classification
PM	Particulate matter
IARC	International Agency for Research on Cancer
ROS	Reactive oxygen species
INE	Instituto Nacional de Estadística (National Statistics Institute)
RETI-SEHOP	Registro Español de Tumores Infantiles-Sociedad Española de Hematología y Oncología Pediátrica (Spanish Childhood Tumor Registry-Spanish Society of Pediatric Hematology and Oncology)
ICCC-3	Classification of Childhood Cancer, Third Edition
OR	Odds ratio
CI	Confidence interval
GAM	Generalized additive models
UFPs	Ultrafine particles
SES	Socio-economic status
WHO	World Health Organization
